# Synthesis, crystal structure and Hirshfeld surface analysis of a coordination compound of silver nitrate with 2-amino­benzoxazole

**DOI:** 10.1107/S2056989025010254

**Published:** 2025-11-25

**Authors:** Surayyo Razzoqova, Sojida Sadullayeva, Sirojiddin Erkinov, Batirbay Torambetov, Guloy Alieva, Zukhra Yakhshieva, Jamshid Ashurov, Shakhnoza Kadirova

**Affiliations:** a National University of Uzbekistan named after Mirzo Ulugbek 4 University St Tashkent 100174 Uzbekistan; b Khorezm Mamun branch of Uzbekistan Academy of Sciences, 1, Markaz St., Khiva, 220900, Uzbekistan; c Physical and Material Chemistry Division CSIR-National Chemical Laboratory,Pune 411008 India; d Jizzakh State Pedagogical University, 4 Sh. Rashidiv St., Jizzakh, 130100, Uzbekistan; e Institute of Bioorganic Chemistry, Academy of Sciences of Uzbekistan, M. Ulugbek, St, 83, Tashkent, 100125, Uzbekistan; Universidad de la República, Uruguay

**Keywords:** crystal structure, cadmium complex, 2-amino­benzoxazole, linear geometry

## Abstract

The coordination complex of 2-amino­benzaxole (2AB) with silver(I), namely, bis(2-aminobenzoxazole-κ*N*)silver(I) nitrate–bis(2-aminobenzoxazole (1/2), [Ag(C_7_H_6_N_2_O)_2_]NO_3_·2C_7_H_6_N_2_O or [Ag(2AB)_2_]NO_3_·(2AB)_2_, was synthesized from ethanol solutions of AgNO_3_ and 2AB. The asymmetric unit contains one mol­ecule of [Ag(2AB)_2_]NO_3_·(2AB)_2_, The central silver(I) atom is coordinated by two nitro­gen donor atoms from 2-amino­benzaxazole ligands in an N_2_ coordination set while another two 2-amino­benzaxazole ligands and one nitrate anion remain uncoordinated. The crystal structure features several intra­molecular N—H⋯O and N—H⋯N hydrogen-bonding inter­actions as well as C—H⋯π, Ag⋯π and π–π inter­actions between adjacent AB ligands. Hirshfeld surface analysis and two-dimensional fingerprint plots were used to investigate the inter­molecular inter­actions.

## Abstract 2

In the complex [Ag(2AB)_2_]NO_3_·(2AB)_2_ (2AB: 2-amino­benzoxazole), the central silver atom is coordinated monodentately by two 2AB ligands forming a linear geometry. The extended structure features N—H⋯N, N—H⋯π and π–π inter­actions.

## 1. Chemical context

The benzoxazole framework has been explored for its anti­tubercular potential since the early 19th century (Wagner & Vonderbank, 1949 ▸; Šlachtová & Brulíková, 2018 ▸). In recent decades, 2-amino­benzoxazole (2AB) has attracted considerable attention due to its structural versatility and broad spectrum of applications in pharmaceuticals because of its anti­bacterial (Paramashivappa *et al.*, 2003 ▸), anti-inflammatory (Parlapalli & Manda, 2017 ▸), anti­tumour (Imaizumi *et al.*, 2020 ▸), anti­microbial (Erol *et al.*, 2022 ▸;), analgesic (Ali *et al.*, 2022 ▸; Sattar *et al.*, 2020 ▸) and fungicidal activities (Fan *et al.*, 2022 ▸), as well as in agrochemicals and materials science (Potashman *et al.*, 2007 ▸). Substituents at the 2- and 5-positions of the benzene ring have been found to significantly enhance biological activity, particularly anti­tubercular effects (Manna & Agrawal, 2010 ▸; Sharma *et al.*, 2011 ▸; Shaharyar *et al.*, 2006 ▸). Moreover, 2AB has emerged as a promising candidate in anti­viral drug development, as it acts as a ligand for the inter­nal ribosome entry site (IRES) RNA of the hepatitis C virus (Rynearson *et al.*, 2014 ▸). In this study, we present the synthesis of a silver(I) coordination complex with 2AB, along with its crystal structure, supra­mol­ecular characteristics, and Hirshfeld surface analysis.
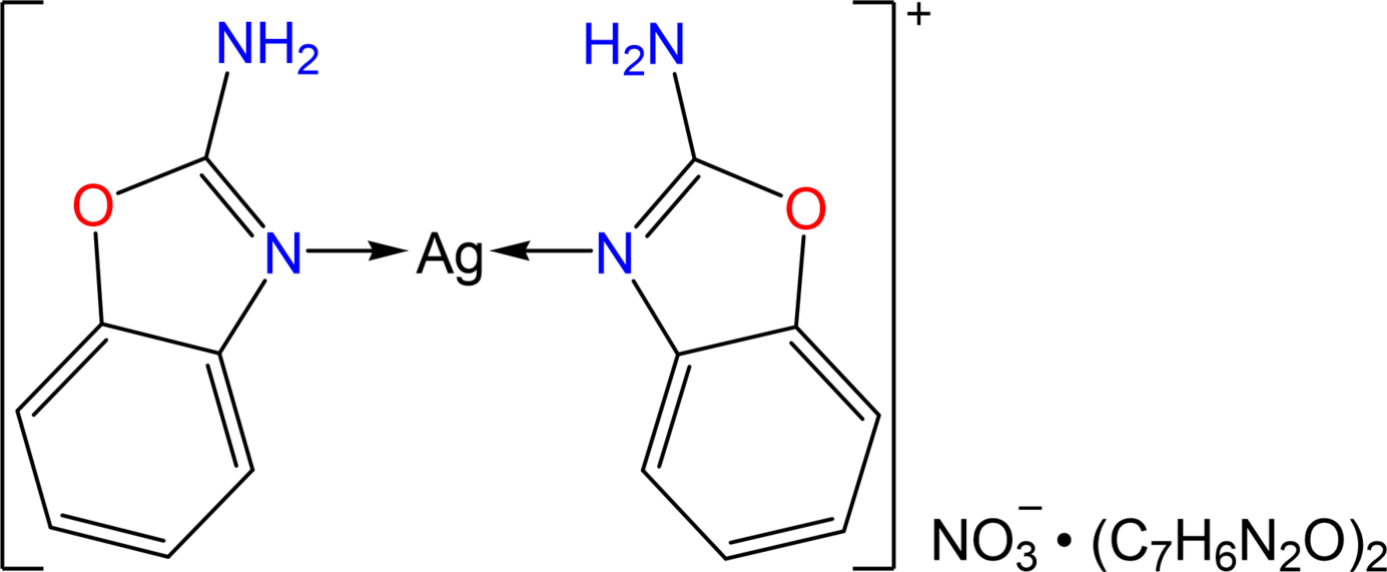


## 2. Structural commentary

**Figure 1:**
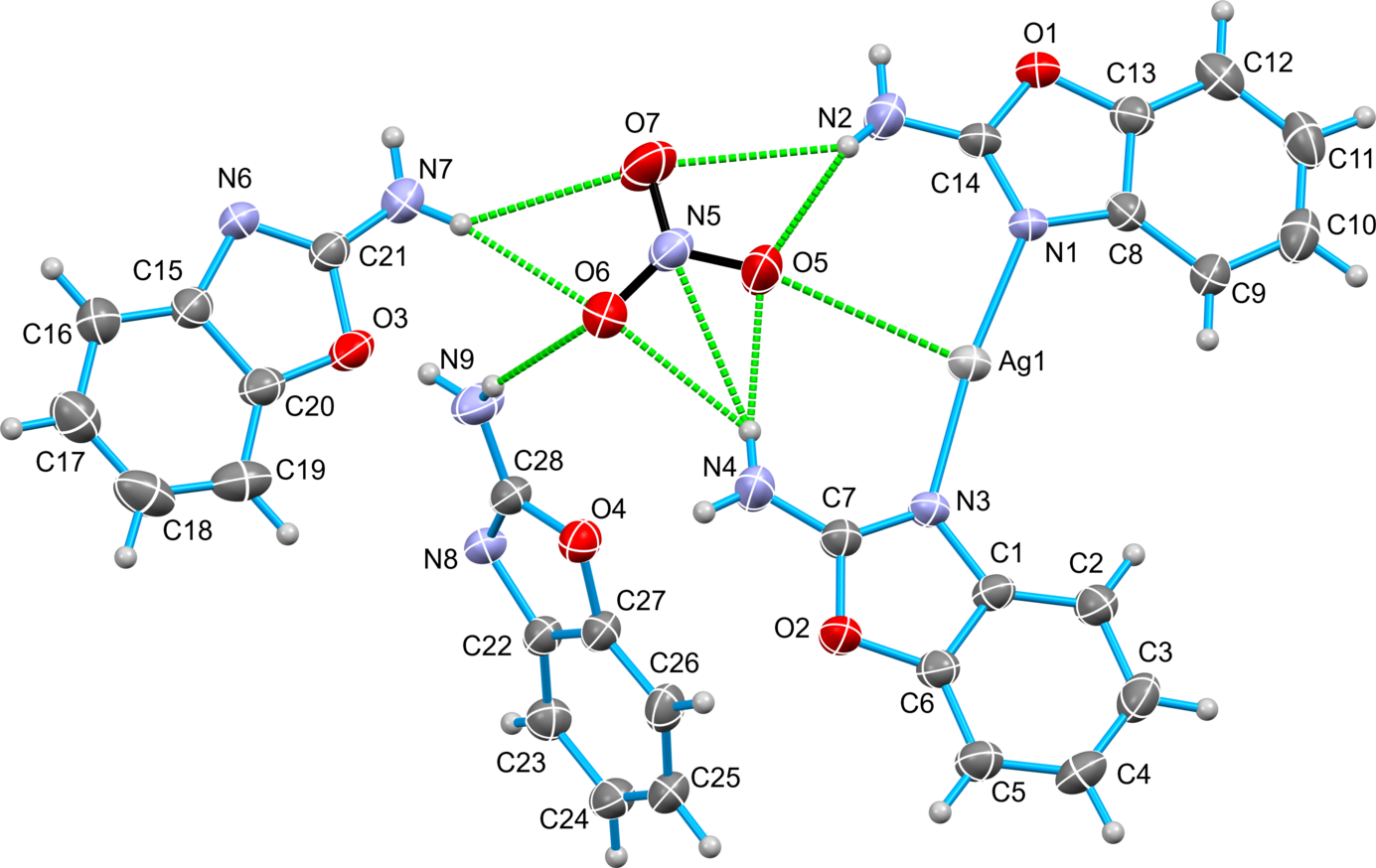
[Ag(2AB)_2_]NO_3_·(2AB)_2_ with displacement ellipsoids drawn at the 30% ellipsoid probability level showing the atom labelling. Hydrogen atoms are represented as small spheres with arbitrary radii and hydrogen bonds are indicated by dashed lines.

The asymmetric unit of the synthesized complex consists of a single [Ag(2AB)_2_]NO_3_·(2AB)_2_ mol­ecule. The central silver(I) atom is coordinated by two nitro­gen donor atoms from 2-amino­benzoxazole ligands, forming an N_2_ coordination set in a linear geometry while another two 2-amino­benzaxazole ligands and one nitrate anion remain uncoordinated (see Fig. 1 ▸). Each 2AB ligand binds in a monodentate fashion *via* its neutral nitro­gen atom, exhibiting Ag—N bond lengths of 2.110 (5) and 2.116 (5) Å. The dihedral angle between the two oppositely coordinated 2-amino­benzoxazole ligands is 2.55 (7)°.

## 3. Supra­molecular features

**Figure 2:**
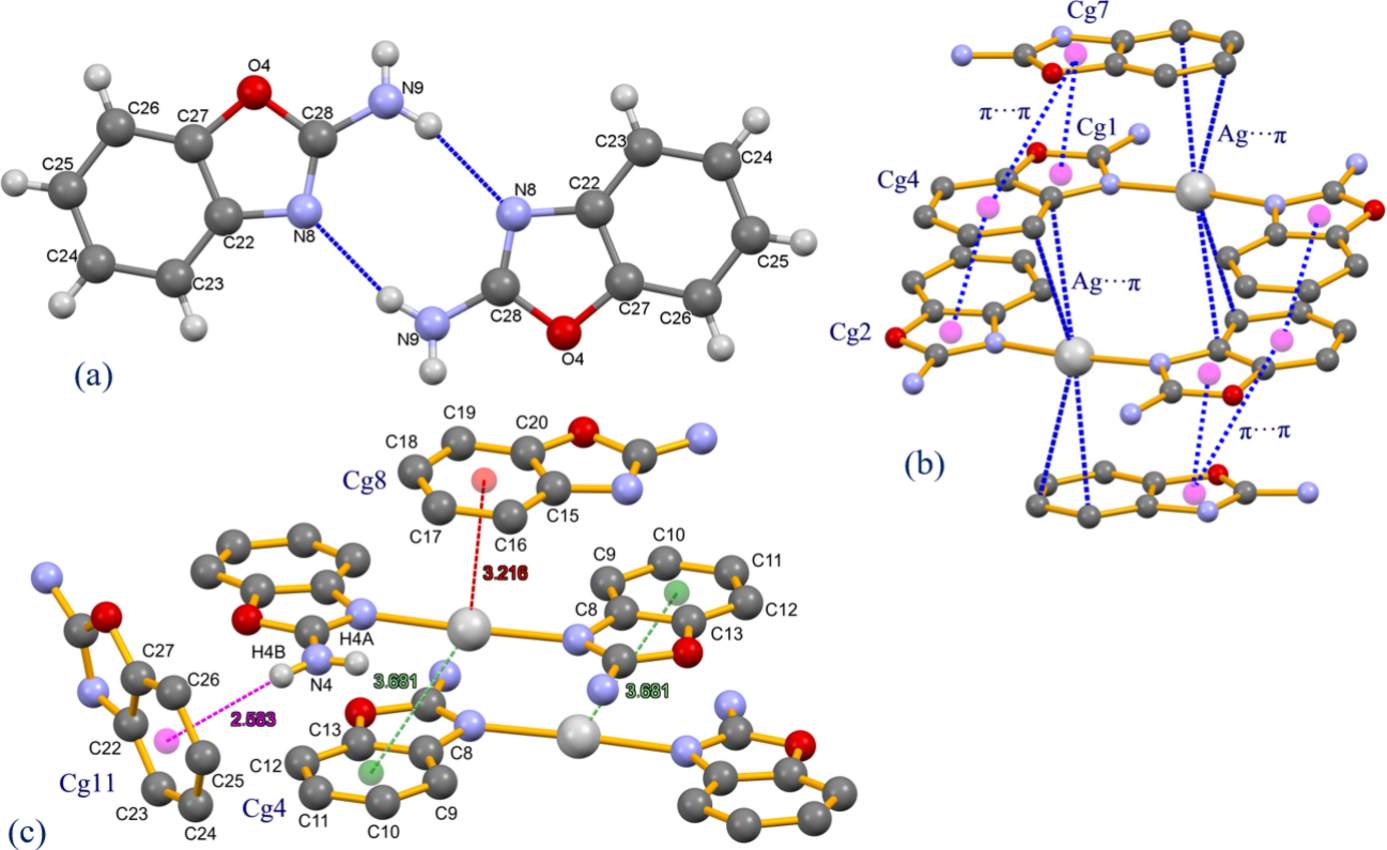
The packing of [Ag(2AB)_2_]NO_3_·(2AB)_2_ showing N—H⋯O, N—H⋯N, N—H⋯π, Ag⋯π, and π–π inter­actions.

Hydrogen-bonding inter­actions occur between the components of the title complex. In particular, the amino groups of the two coordinated 2-amino­benzoxazole ligands inter­act with an oxygen and nitro­gen atom of the nitrate anion through N—H⋯O and N—H⋯N inter­actions, while both of the uncoordinated 2-amino­benzoxazole ligands also form an N—H⋯O hydrogen bond with the nitrate anion (Table 1 ▸). Several N—H⋯N hydrogen bonds also occur. There is also an N—H⋯π inter­action between the amino group and the six-membered aromatic ring of the 2-amino­benzoxazole ligand, N4—H4*B*⋯*Cg*11 (Fig. 2 ▸, Table 1 ▸). In addition both of the coordinated 2AB ligands participate in π–π inter­actions [*Cg*1⋯*Cg*7^iv^ = 3.584 (4) Å, dihedral angle = 6.7 (4)°; *Cg*2⋯*Cg*4^v^ = 3.609 (4) Å, dihedral angle = 2.5 (4)°; *Cg*4⋯*Cg*7^iv^ = 3.953 (4) Å, dihedral angle = 6.4 (4)°; where *Cg*1, *Cg*2, *Cg*4 and*Cg*7 are the centroids of the O1/C13/C8/N1/C14, O2/C6/C1/N3/C7, C8–13 and O3/C20/C15/N6/C21 rings, respectively; symmetry codes: (iv) *x*, *y* − 1, *z*; (v) 1 − *x*, −*y*, −*z*]. η^2^ Ag⋯π inter­actions are also observed involving adjacent carbon atoms of two phenyl rings. In the first ring, the Ag1⋯C8 and Ag1⋯C9 distances are 3.411 (6) and 3.186 (7) Å, respectively, while in the second ring, the Ag1⋯C16 and Ag1⋯C17 distances are 3.418 (9) and 3.345 (9) Å, respectively.

## 4. Hirshfeld Surface Analysis

**Figure 3:**
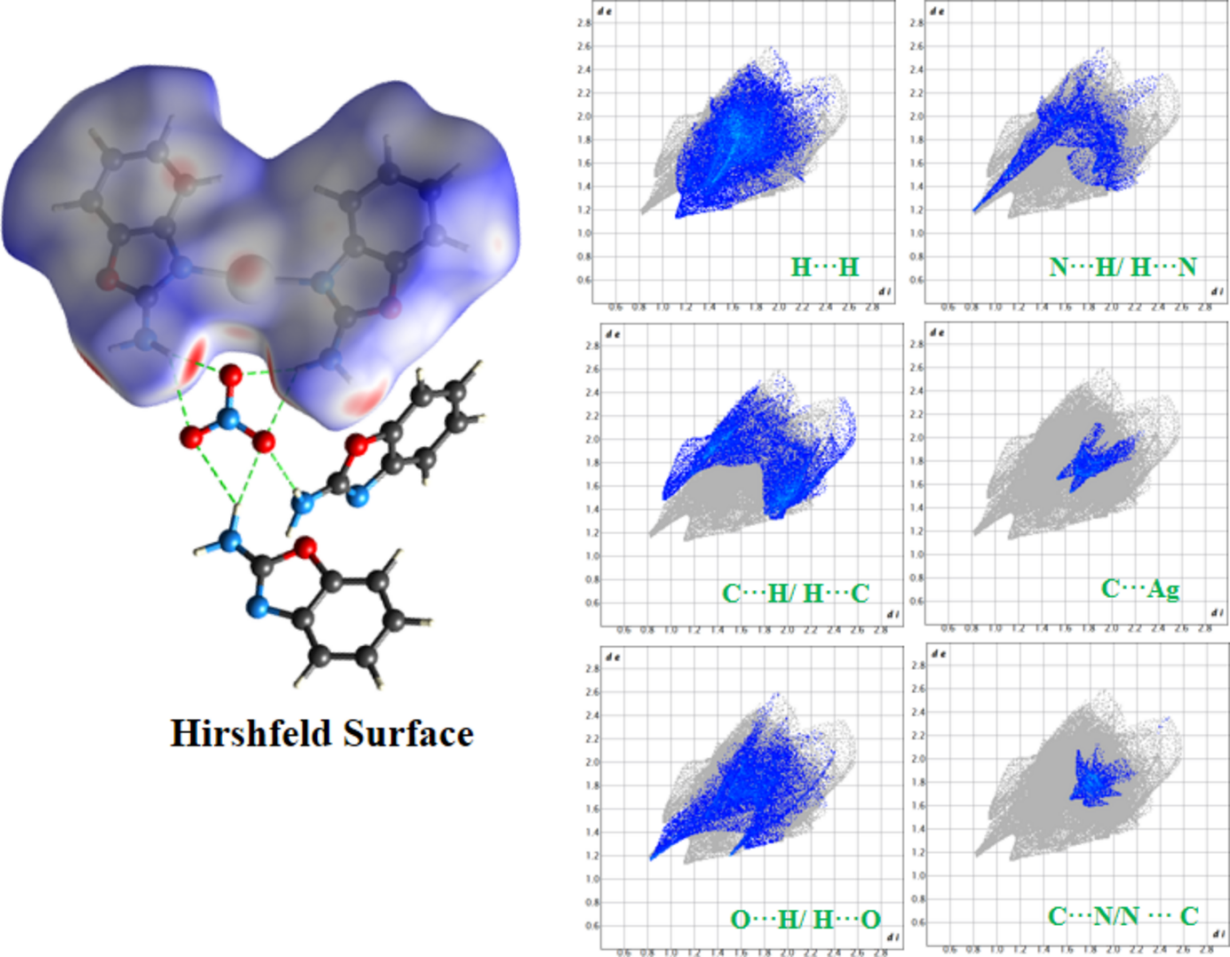
The Hirshfeld surface and corresponding two-dimensional fingerprint plots illustrating the contributions of different inter­molecular contacts.

A Hirshfeld surface (HS) analysis (Spackman & Jayatilaka, 2009 ▸) was performed and the two-dimensional fingerprint plots (Spackman & McKinnon, 2002 ▸) were generated using *CrystalExplorer* (Spackman *et al.*, 2021 ▸) to qu­antify the inter­molecular inter­actions (Fig. 3 ▸). The red spots on the HS indicate the presence of close inter­molecular N—H⋯O and N—H⋯N inter­actions. The fingerprint plots shows that H⋯H (31.50%), C⋯H/H⋯C (19.60%), O⋯H/H⋯O (17.2%), N⋯H/H⋯N (9.60%), C⋯C (5.30%), C⋯N/N⋯C (4.40%), C⋯O/O⋯C (3.90%), and Ag⋯C/C⋯Ag (4.20%) are the major inter­actions contributing ∼95.7% to the HS with minor inter­actions contributing less than 5%.

## 5. Database survey

A survey of the Cambridge Structural Database (CSD, Version 5.46, November 2024; Groom *et al.*, 2016 ▸) identified 18 crystal structures of 2-amino­benzoxazole (2AB) derivatives. Among them, only three structures (DIWPIM; Razzoqova *et al.*, 2023 ▸, MUYZEP; Razzoqova *et al.*, 2025 ▸, QALXIL; Decken & Gossage, 2005 ▸) were found for the 2-amino­benzoxazole moiety. Among these, one structure involves a zinc coordination complex (QALXIL), and two structures involve cadmium complexes (DIWPIM, MUYZEP). In the zinc complex QALXIL, the Zn^II^ centre adopts a distorted tetra­hedral geometry, coordinating two 2AB ligands *via* their aromatic nitro­gen atoms, along with two chloride ligands. The cadmium complex [Cd(2AB)_2_(CH3COO)_2_] (DIWPIM) features a Cd^II^ ion coordinated by two 2AB ligands and two acetate ligands, binding in both monodentate and bidentate modes, resulting in a distorted octa­hedral coordination environment with an N_2_O_4_ donor set. In the complex [Cd(2AB)_2_(NO_3_)_2_] (MUYZEP), the cadmium(II) ion is coordinated by four 2AB ligands and two nitrate ions, forming a distorted octa­hedral geometry with an N_4_O_2_ coordination sphere.

## 6. Synthesis and crystallization

AgNO_3_ (0.170 g, 1 mmol) and 2AB (0.268 g, 2 mmol) were dissolved separately in ethanol (5 ml), mixed together and stirred for 2 h. The obtained colourless solution was filtered and left for crystallization. Single crystals of the complex [Ag(AB)_2_](NO_3_)(AB)_2_ suitable for X-ray analysis were obtained by slow evaporation of the solution over a period of 15d.

## 7. Refinement

Crystal data, data collection, and structure refinement details are summarized in Table 2 ▸. All hydrogen atoms were located from difference-Fourier maps and refined isotropically; DFIX restraints were applied to the N—H bond lengths.

## Supplementary Material

Crystal structure: contains datablock(s) I. DOI: 10.1107/S2056989025010254/oo2013sup1.cif

Structure factors: contains datablock(s) I. DOI: 10.1107/S2056989025010254/oo2013Isup2.hkl

CCDC reference: 2502918

Additional supporting information:  crystallographic information; 3D view; checkCIF report

## Figures and Tables

**Table 1:** Hydrogen-bond geometry (Å, °) *Cg*11 is the centroid of the C22–C27 ring.

*D*—H⋯*A*	*D*—H	H⋯*A*	*D*⋯*A*	*D*—H⋯*A*
N2—H2*A*⋯N6^i^	0.84 (2)	2.15 (5)	2.915 (8)	151 (7)
N2—H2*B*⋯O5	0.83 (2)	2.15 (3)	2.937 (10)	158 (8)
N2—H2*B*⋯O7	0.83 (2)	2.65 (7)	3.271 (10)	133 (8)
N4—H4*A*⋯O5	0.85 (2)	2.14 (4)	2.937 (9)	157 (9)
N4—H4*A*⋯O6	0.85 (2)	2.46 (6)	3.084 (8)	131 (6)
N4—H4*A*⋯N5	0.85 (2)	2.64 (4)	3.434 (8)	156 (7)
N7—H7*A*⋯O7^i^	0.84 (2)	2.17 (2)	3.012 (9)	175 (7)
N7—H7*B*⋯O6	0.85 (2)	2.24 (3)	3.069 (8)	165 (8)
N9—H9*A*⋯N8^ii^	0.85 (2)	2.18 (3)	2.997 (9)	161 (9)
N9—H9*B*⋯O6	0.85 (2)	2.28 (5)	3.041 (10)	149 (9)
N4—H4*B*⋯*Cg*11^iii^	0.85 (4)	2.58 (5)	3.403 (7)	162 (7)

Symmetry codes: (i) 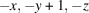; (ii) 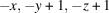; (iii) 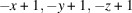.

**Table 2:** Experimental details

Crystal data	
Chemical formula	[Ag(C_7_H_6_N_2_O)_2_]NO_3_·2C_7_H_6_N_2_O
*M*_r_	706.43
Crystal system, space group	Triclinic, *P* 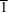
Temperature (K)	293
*a*, *b*, *c* (Å)	10.6356 (3), 11.2202 (5), 12.3475 (3)
α, β, γ (°)	92.459 (3), 94.903 (2), 98.682 (3)
*V* (Å^3^)	1448.91 (8)
*Z*	2
Radiation type	Cu *K*α
μ (mm^−1^)	6.13
Crystal size (mm)	0.11 × 0.09 × 0.08

Data collection	
Diffractometer	XtaLAB Synergy, Single source at home/near, H
Absorption correction	Multi-scan (*CrysAlis PRO* ; Rigaku OD, 2020 ▸)
*T*_min_, *T*_max_	0.332, 0.642
No. of measured, independent and observed [*I* > 2σ(*I*)] reflections	14506, 5559, 4536
*R*_int_	0.074
(sin θ/λ)_max_ (Å^−1^)	0.615

Refinement	
*R*[*F*^2^ > 2σ(*F*^2^)], *wR*(*F*^2^), *S*	0.070, 0.235, 1.12
No. of reflections	5559
No. of parameters	430
No. of restraints	12
H-atom treatment	H atoms treated by a mixture of independent and constrained refinement
Δρ_max_, Δρ_min_ (e Å^−3^)	1.62, −1.69

Computer programs: *CrysAlis PRO*(Rigaku OD, 2020 ▸), *SHELXT* (Sheldrick, 2015*a* ▸), *SHELXL2019/2* (Sheldrick, 2015*b* ▸) and *OLEX2* (Dolomanov *et al.*, 2009 ▸).

## supplementary crystallographic information

### Bis(2-aminobenzoxazole-κN)silver(I) nitrate–bis(2-aminobenzoxazole (1/2) Crystal data

**Table:**

[Ag(C_7_H_6_N_2_O)_2_]NO_3_·2C_7_H_6_N_2_O	*Z* = 2
*M**_r_* = 706.43	*F*(000) = 716
Triclinic, *P*1	*D*_x_ = 1.619 Mg m^−^^3^
*a* = 10.6356 (3) Å	Cu *K*α radiation, λ = 1.54184 Å
*b* = 11.2202 (5) Å	Cell parameters from 5786 reflections
*c* = 12.3475 (3) Å	θ = 3.6–71.3°
α = 92.459 (3)°	µ = 6.13 mm^−^^1^
β = 94.903 (2)°	*T* = 293 K
γ = 98.682 (3)°	Block, colourless
*V* = 1448.91 (8) Å^3^	0.11 × 0.09 × 0.08 mm

### Bis(2-aminobenzoxazole-κN)silver(I) nitrate–bis(2-aminobenzoxazole (1/2) Data collection

**Table:**

XtaLAB Synergy, Single source at home/near, H diffractometer	5559 independent reflections
Radiation source: micro-focus sealed X-ray tube	4536 reflections with *I* > 2σ(*I*)
Detector resolution: 10.0000 pixels mm^-1^	*R*_int_ = 0.074
ω scans	θ_max_ = 71.6°, θ_min_ = 3.6°
Absorption correction: multi-scan (CrysAlisPro ; Rigaku OD, 2020)	*h* = −13→10
*T*_min_ = 0.332, *T*_max_ = 0.642	*k* = −13→13
14506 measured reflections	*l* = −15→15

### Bis(2-aminobenzoxazole-κN)silver(I) nitrate–bis(2-aminobenzoxazole (1/2) Refinement

**Table:**

Refinement on *F*^2^	Primary atom site location: dual
Least-squares matrix: full	Secondary atom site location: difference Fourier map
*R*[*F*^2^ > 2σ(*F*^2^)] = 0.070	Hydrogen site location: mixed
*wR*(*F*^2^) = 0.235	H atoms treated by a mixture of independent and constrained refinement
*S* = 1.12	*w* = 1/[σ^2^(*F*_o_^2^) + (0.1293*P*)^2^ + 1.0923*P*] where *P* = (*F*_o_^2^ + 2*F*_c_^2^)/3
5559 reflections	(Δ/σ)_max_ < 0.001
430 parameters	Δρ_max_ = 1.62 e Å^−^^3^
12 restraints	Δρ_min_ = −1.69 e Å^−^^3^

### Bis(2-aminobenzoxazole-κN)silver(I) nitrate–bis(2-aminobenzoxazole (1/2) Special details

**Table:**

Geometry. All esds (except the esd in the dihedral angle between two l.s. planes) are estimated using the full covariance matrix. The cell esds are taken into account individually in the estimation of esds in distances, angles and torsion angles; correlations between esds in cell parameters are only used when they are defined by crystal symmetry. An approximate (isotropic) treatment of cell esds is used for estimating esds involving l.s. planes.

### Bis(2-aminobenzoxazole-κN)silver(I) nitrate–bis(2-aminobenzoxazole (1/2) Fractional atomic coordinates and isotropic or equivalent isotropic displacement parameters (Å^2^)

**Table:**

	*x*	*y*	*z*	*U*_iso_*/*U*_eq_	
Ag1	0.42768 (4)	0.08778 (4)	0.14010 (4)	0.0590 (2)	
O1	0.1314 (4)	−0.0952 (4)	−0.0925 (4)	0.0592 (11)	
O2	0.6801 (5)	0.3272 (4)	0.3700 (4)	0.0638 (12)	
N1	0.3036 (5)	−0.0216 (5)	0.0205 (4)	0.0484 (11)	
N2	0.1515 (7)	0.1029 (6)	−0.0354 (7)	0.0746 (17)	
H2A	0.089 (6)	0.090 (7)	−0.083 (6)	0.090*	
H2B	0.193 (7)	0.170 (4)	−0.016 (7)	0.090*	
N3	0.5661 (5)	0.1799 (5)	0.2604 (4)	0.0512 (11)	
N4	0.4995 (7)	0.3708 (6)	0.2761 (6)	0.0752 (18)	
H4A	0.429 (5)	0.350 (6)	0.238 (7)	0.090*	
H4B	0.523 (7)	0.445 (3)	0.297 (7)	0.090*	
C1	0.6719 (6)	0.1359 (6)	0.3110 (5)	0.0521 (13)	
C2	0.7103 (7)	0.0237 (6)	0.3025 (6)	0.0627 (16)	
H2	0.662384	−0.040164	0.259658	0.075*	
C3	0.8249 (8)	0.0118 (7)	0.3617 (7)	0.075 (2)	
H3	0.854698	−0.061943	0.358073	0.090*	
C4	0.8953 (7)	0.1069 (8)	0.4257 (7)	0.076 (2)	
H4	0.972577	0.096078	0.462218	0.092*	
C5	0.8547 (7)	0.2175 (7)	0.4370 (6)	0.0670 (17)	
H5	0.900920	0.281153	0.481321	0.080*	
C6	0.7402 (6)	0.2273 (6)	0.3778 (5)	0.0549 (14)	
C7	0.5768 (6)	0.2919 (6)	0.2976 (5)	0.0543 (14)	
C8	0.3061 (6)	−0.1432 (6)	−0.0033 (5)	0.0531 (14)	
C9	0.3949 (7)	−0.2174 (6)	0.0292 (6)	0.0612 (16)	
H9	0.467054	−0.188776	0.076390	0.073*	
C10	0.3695 (9)	−0.3366 (7)	−0.0128 (7)	0.078 (2)	
H10	0.425922	−0.389097	0.007756	0.093*	
C11	0.2646 (10)	−0.3792 (7)	−0.0833 (8)	0.083 (2)	
H11	0.251777	−0.459468	−0.109480	0.099*	
C12	0.1768 (8)	−0.3055 (7)	−0.1166 (6)	0.0702 (19)	
H12	0.105417	−0.333766	−0.164773	0.084*	
C13	0.2017 (6)	−0.1878 (6)	−0.0740 (5)	0.0574 (14)	
C14	0.1975 (6)	0.0008 (6)	−0.0326 (5)	0.0535 (14)	
O3	0.2193 (5)	0.7580 (5)	0.2610 (4)	0.0659 (12)	
N6	0.0707 (5)	0.8530 (5)	0.1741 (5)	0.0586 (13)	
N7	0.0691 (7)	0.6442 (6)	0.1405 (5)	0.0709 (16)	
H7A	0.013 (6)	0.643 (7)	0.088 (5)	0.085*	
H7B	0.125 (6)	0.598 (6)	0.140 (6)	0.085*	
C15	0.1506 (7)	0.9375 (7)	0.2480 (5)	0.0607 (16)	
C16	0.1538 (8)	1.0573 (8)	0.2688 (7)	0.076 (2)	
H16	0.095172	1.098802	0.232582	0.091*	
C17	0.2475 (9)	1.1161 (9)	0.3460 (8)	0.083 (2)	
H17	0.251395	1.198372	0.362370	0.100*	
C18	0.3339 (9)	1.0549 (11)	0.3983 (7)	0.090 (3)	
H18	0.394775	1.096878	0.450410	0.108*	
C19	0.3348 (8)	0.9332 (10)	0.3771 (7)	0.085 (2)	
H19	0.394560	0.892073	0.412464	0.101*	
C20	0.2420 (7)	0.8774 (7)	0.3007 (6)	0.0622 (16)	
C21	0.1159 (6)	0.7536 (6)	0.1864 (5)	0.0552 (14)	
O4	0.2916 (4)	0.4227 (4)	0.4621 (4)	0.0620 (11)	
N8	0.1235 (5)	0.4191 (5)	0.5619 (4)	0.0558 (12)	
N9	0.1122 (7)	0.4853 (8)	0.3811 (6)	0.0777 (19)	
H9A	0.040 (4)	0.509 (8)	0.382 (7)	0.093*	
H9B	0.160 (6)	0.509 (8)	0.332 (6)	0.093*	
C22	0.2243 (6)	0.3788 (5)	0.6238 (5)	0.0532 (13)	
C23	0.2349 (7)	0.3414 (6)	0.7274 (6)	0.0624 (16)	
H23	0.166796	0.337238	0.770247	0.075*	
C24	0.3519 (7)	0.3095 (7)	0.7669 (6)	0.0674 (18)	
H24	0.361718	0.285213	0.837797	0.081*	
C25	0.4530 (7)	0.3132 (7)	0.7036 (6)	0.0672 (18)	
H25	0.529411	0.291607	0.732625	0.081*	
C26	0.4424 (7)	0.3483 (7)	0.5981 (7)	0.0649 (17)	
H26	0.509288	0.350226	0.554071	0.078*	
C27	0.3262 (6)	0.3804 (6)	0.5617 (6)	0.0566 (14)	
C28	0.1690 (6)	0.4432 (6)	0.4704 (6)	0.0589 (15)	
O5	0.3017 (6)	0.3107 (6)	0.0929 (6)	0.093 (2)	
O6	0.2585 (6)	0.4664 (5)	0.1816 (5)	0.0798 (15)	
O7	0.1249 (6)	0.3756 (7)	0.0546 (7)	0.109 (2)	
N5	0.2266 (6)	0.3839 (6)	0.1100 (5)	0.0657 (15)	

### Bis(2-aminobenzoxazole-κN)silver(I) nitrate–bis(2-aminobenzoxazole (1/2) Atomic displacement parameters (Å^2^)

**Table:**

	*U*^11^	*U*^22^	*U*^33^	*U*^12^	*U*^13^	*U*^23^
Ag1	0.0569 (4)	0.0592 (3)	0.0571 (3)	0.0089 (2)	−0.0119 (2)	−0.0049 (2)
O1	0.053 (2)	0.066 (3)	0.056 (2)	0.011 (2)	−0.0104 (19)	−0.004 (2)
O2	0.060 (3)	0.054 (2)	0.072 (3)	0.010 (2)	−0.019 (2)	−0.010 (2)
N1	0.046 (3)	0.051 (3)	0.047 (3)	0.011 (2)	−0.010 (2)	−0.004 (2)
N2	0.073 (4)	0.058 (3)	0.092 (5)	0.024 (3)	−0.012 (3)	0.000 (3)
N3	0.048 (3)	0.049 (3)	0.053 (3)	0.004 (2)	−0.011 (2)	−0.004 (2)
N4	0.069 (4)	0.065 (4)	0.091 (5)	0.026 (3)	−0.015 (3)	−0.015 (3)
C1	0.045 (3)	0.055 (3)	0.054 (3)	0.007 (2)	−0.006 (2)	0.006 (3)
C2	0.070 (4)	0.055 (4)	0.062 (4)	0.012 (3)	−0.008 (3)	0.003 (3)
C3	0.081 (5)	0.067 (4)	0.083 (5)	0.032 (4)	0.003 (4)	0.015 (4)
C4	0.060 (4)	0.089 (6)	0.081 (5)	0.023 (4)	−0.012 (4)	0.017 (4)
C5	0.059 (4)	0.068 (4)	0.070 (4)	0.008 (3)	−0.012 (3)	0.002 (3)
C6	0.055 (3)	0.057 (4)	0.051 (3)	0.009 (3)	−0.003 (3)	0.005 (3)
C7	0.051 (3)	0.052 (3)	0.058 (3)	0.008 (3)	−0.005 (3)	−0.001 (3)
C8	0.059 (4)	0.051 (3)	0.048 (3)	0.010 (3)	0.002 (3)	−0.001 (2)
C9	0.066 (4)	0.060 (4)	0.060 (4)	0.022 (3)	0.001 (3)	0.002 (3)
C10	0.091 (6)	0.065 (5)	0.086 (5)	0.032 (4)	0.016 (5)	0.016 (4)
C11	0.114 (7)	0.051 (4)	0.085 (6)	0.012 (4)	0.018 (5)	−0.009 (4)
C12	0.077 (5)	0.070 (5)	0.060 (4)	0.000 (4)	0.011 (3)	−0.010 (3)
C13	0.058 (4)	0.060 (4)	0.052 (3)	0.007 (3)	−0.001 (3)	0.000 (3)
C14	0.048 (3)	0.058 (3)	0.051 (3)	0.006 (3)	−0.010 (2)	−0.003 (3)
O3	0.062 (3)	0.081 (3)	0.059 (3)	0.030 (2)	−0.003 (2)	0.012 (2)
N6	0.053 (3)	0.067 (3)	0.058 (3)	0.022 (3)	−0.009 (2)	−0.004 (2)
N7	0.078 (4)	0.067 (4)	0.071 (4)	0.027 (3)	−0.003 (3)	0.005 (3)
C15	0.063 (4)	0.072 (4)	0.051 (3)	0.024 (3)	0.004 (3)	0.002 (3)
C16	0.076 (5)	0.078 (5)	0.074 (5)	0.023 (4)	0.001 (4)	−0.009 (4)
C17	0.083 (6)	0.077 (5)	0.084 (6)	0.002 (4)	0.006 (4)	−0.014 (4)
C18	0.068 (5)	0.120 (8)	0.072 (5)	−0.009 (5)	0.001 (4)	−0.021 (5)
C19	0.064 (5)	0.119 (8)	0.066 (5)	0.008 (5)	−0.009 (4)	0.016 (5)
C20	0.058 (4)	0.079 (5)	0.051 (3)	0.020 (3)	−0.002 (3)	0.006 (3)
C21	0.050 (3)	0.067 (4)	0.052 (3)	0.021 (3)	0.002 (3)	0.004 (3)
O4	0.060 (3)	0.073 (3)	0.057 (2)	0.023 (2)	0.006 (2)	0.003 (2)
N8	0.047 (3)	0.063 (3)	0.056 (3)	0.013 (2)	−0.007 (2)	0.000 (2)
N9	0.075 (4)	0.103 (5)	0.060 (4)	0.031 (4)	−0.002 (3)	0.018 (3)
C22	0.049 (3)	0.050 (3)	0.060 (3)	0.012 (2)	−0.004 (3)	0.001 (3)
C23	0.056 (4)	0.064 (4)	0.065 (4)	0.008 (3)	−0.001 (3)	0.001 (3)
C24	0.074 (4)	0.064 (4)	0.064 (4)	0.018 (3)	−0.011 (3)	0.007 (3)
C25	0.067 (4)	0.066 (4)	0.071 (4)	0.028 (3)	−0.010 (3)	0.000 (3)
C26	0.054 (4)	0.067 (4)	0.078 (5)	0.023 (3)	0.009 (3)	0.000 (3)
C27	0.057 (4)	0.052 (3)	0.062 (4)	0.016 (3)	−0.004 (3)	0.003 (3)
C28	0.058 (4)	0.058 (4)	0.061 (4)	0.017 (3)	−0.009 (3)	0.003 (3)
O5	0.080 (4)	0.077 (4)	0.123 (5)	0.039 (3)	−0.028 (3)	−0.023 (3)
O6	0.089 (4)	0.080 (4)	0.074 (3)	0.033 (3)	−0.005 (3)	−0.005 (3)
O7	0.077 (4)	0.098 (5)	0.145 (6)	0.023 (3)	−0.045 (4)	−0.002 (4)
N5	0.060 (3)	0.066 (4)	0.072 (4)	0.020 (3)	−0.006 (3)	0.010 (3)

### Bis(2-aminobenzoxazole-κN)silver(I) nitrate–bis(2-aminobenzoxazole (1/2) Geometric parameters (Å, º)

**Table:**

Ag1—N1	2.110 (5)	O3—C20	1.386 (10)
Ag1—N3	2.116 (5)	N6—C21	1.289 (8)
O1—C14	1.347 (8)	N6—C15	1.415 (9)
O1—C13	1.384 (8)	N7—C21	1.335 (10)
O2—C7	1.353 (8)	N7—H7A	0.84 (2)
O2—C6	1.375 (8)	N7—H7B	0.85 (2)
N1—C14	1.319 (8)	C15—C16	1.352 (11)
N1—C8	1.388 (8)	C15—C20	1.397 (9)
N2—C14	1.313 (9)	C16—C17	1.386 (12)
N2—H2A	0.84 (2)	C16—H16	0.9300
N2—H2B	0.83 (2)	C17—C18	1.365 (14)
N3—C7	1.305 (8)	C17—H17	0.9300
N3—C1	1.406 (8)	C18—C19	1.382 (15)
N4—C7	1.316 (9)	C18—H18	0.9300
N4—H4A	0.85 (2)	C19—C20	1.363 (11)
N4—H4B	0.86 (2)	C19—H19	0.9300
C1—C6	1.363 (9)	O4—C28	1.370 (8)
C1—C2	1.383 (10)	O4—C27	1.378 (8)
C2—C3	1.394 (10)	N8—C28	1.289 (9)
C2—H2	0.9300	N8—C22	1.405 (7)
C3—C4	1.382 (12)	N9—C28	1.349 (9)
C3—H3	0.9300	N9—H9A	0.85 (2)
C4—C5	1.380 (11)	N9—H9B	0.85 (2)
C4—H4	0.9300	C22—C23	1.364 (9)
C5—C6	1.388 (9)	C22—C27	1.379 (9)
C5—H5	0.9300	C23—C24	1.400 (10)
C8—C13	1.369 (10)	C23—H23	0.9300
C8—C9	1.395 (9)	C24—C25	1.378 (11)
C9—C10	1.393 (11)	C24—H24	0.9300
C9—H9	0.9300	C25—C26	1.378 (11)
C10—C11	1.367 (13)	C25—H25	0.9300
C10—H10	0.9300	C26—C27	1.383 (9)
C11—C12	1.385 (13)	C26—H26	0.9300
C11—H11	0.9300	O5—N5	1.253 (8)
C12—C13	1.380 (10)	O6—N5	1.240 (8)
C12—H12	0.9300	O7—N5	1.218 (8)
O3—C21	1.367 (8)		
			
N1—Ag1—N3	172.85 (18)	N1—C14—O1	113.8 (5)
C14—O1—C13	104.7 (5)	C21—O3—C20	103.4 (5)
C7—O2—C6	104.8 (5)	C21—N6—C15	104.2 (5)
C14—N1—C8	105.1 (5)	C21—N7—H7A	115 (6)
C14—N1—Ag1	129.1 (4)	C21—N7—H7B	112 (6)
C8—N1—Ag1	124.9 (4)	H7A—N7—H7B	121 (4)
C14—N2—H2A	105 (6)	C16—C15—C20	120.3 (7)
C14—N2—H2B	125 (6)	C16—C15—N6	131.9 (7)
H2A—N2—H2B	125 (4)	C20—C15—N6	107.8 (6)
C7—N3—C1	105.3 (5)	C15—C16—C17	117.8 (8)
C7—N3—Ag1	127.5 (4)	C15—C16—H16	121.1
C1—N3—Ag1	127.0 (4)	C17—C16—H16	121.1
C7—N4—H4A	121 (5)	C18—C17—C16	120.8 (9)
C7—N4—H4B	120 (5)	C18—C17—H17	119.6
H4A—N4—H4B	119 (4)	C16—C17—H17	119.6
C6—C1—C2	121.3 (6)	C17—C18—C19	122.7 (8)
C6—C1—N3	107.6 (5)	C17—C18—H18	118.6
C2—C1—N3	131.2 (6)	C19—C18—H18	118.6
C1—C2—C3	116.3 (7)	C20—C19—C18	115.3 (8)
C1—C2—H2	121.9	C20—C19—H19	122.3
C3—C2—H2	121.9	C18—C19—H19	122.3
C4—C3—C2	121.5 (7)	C19—C20—O3	129.0 (7)
C4—C3—H3	119.2	C19—C20—C15	123.0 (8)
C2—C3—H3	119.2	O3—C20—C15	108.0 (6)
C5—C4—C3	122.1 (7)	N6—C21—N7	128.0 (6)
C5—C4—H4	118.9	N6—C21—O3	116.5 (6)
C3—C4—H4	118.9	N7—C21—O3	115.3 (6)
C4—C5—C6	115.4 (7)	C28—O4—C27	103.6 (5)
C4—C5—H5	122.3	C28—N8—C22	104.0 (5)
C6—C5—H5	122.3	C28—N9—H9A	122 (6)
C1—C6—O2	108.6 (5)	C28—N9—H9B	117 (6)
C1—C6—C5	123.3 (6)	H9A—N9—H9B	118 (4)
O2—C6—C5	128.0 (6)	C23—C22—C27	119.4 (6)
N3—C7—N4	128.5 (6)	C23—C22—N8	131.8 (6)
N3—C7—O2	113.7 (5)	C27—C22—N8	108.7 (5)
N4—C7—O2	117.8 (6)	C22—C23—C24	117.7 (7)
C13—C8—N1	108.4 (6)	C22—C23—H23	121.2
C13—C8—C9	120.3 (6)	C24—C23—H23	121.2
N1—C8—C9	131.3 (6)	C25—C24—C23	121.8 (7)
C10—C9—C8	116.4 (7)	C25—C24—H24	119.1
C10—C9—H9	121.8	C23—C24—H24	119.1
C8—C9—H9	121.8	C26—C25—C24	121.0 (6)
C11—C10—C9	122.3 (7)	C26—C25—H25	119.5
C11—C10—H10	118.9	C24—C25—H25	119.5
C9—C10—H10	118.9	C25—C26—C27	115.9 (7)
C10—C11—C12	121.5 (7)	C25—C26—H26	122.0
C10—C11—H11	119.2	C27—C26—H26	122.0
C12—C11—H11	119.2	O4—C27—C22	107.9 (5)
C13—C12—C11	116.0 (8)	O4—C27—C26	127.9 (6)
C13—C12—H12	122.0	C22—C27—C26	124.2 (6)
C11—C12—H12	122.0	N8—C28—N9	129.0 (6)
C8—C13—C12	123.5 (7)	N8—C28—O4	115.8 (5)
C8—C13—O1	108.0 (6)	N9—C28—O4	115.2 (6)
C12—C13—O1	128.5 (7)	O7—N5—O6	120.7 (7)
N2—C14—N1	128.5 (6)	O7—N5—O5	120.0 (7)
N2—C14—O1	117.7 (6)	O6—N5—O5	119.3 (6)
			
C7—N3—C1—C6	0.5 (7)	C8—N1—C14—O1	−2.1 (7)
Ag1—N3—C1—C6	176.1 (4)	Ag1—N1—C14—O1	−171.0 (4)
C7—N3—C1—C2	−179.8 (7)	C13—O1—C14—N2	179.8 (7)
Ag1—N3—C1—C2	−4.2 (11)	C13—O1—C14—N1	1.0 (7)
C6—C1—C2—C3	−2.9 (11)	C21—N6—C15—C16	177.0 (8)
N3—C1—C2—C3	177.4 (7)	C21—N6—C15—C20	0.1 (7)
C1—C2—C3—C4	0.4 (12)	C20—C15—C16—C17	−2.1 (12)
C2—C3—C4—C5	1.9 (13)	N6—C15—C16—C17	−178.6 (7)
C3—C4—C5—C6	−1.5 (12)	C15—C16—C17—C18	0.5 (13)
C2—C1—C6—O2	−179.9 (6)	C16—C17—C18—C19	0.9 (14)
N3—C1—C6—O2	−0.2 (7)	C17—C18—C19—C20	−0.7 (13)
C2—C1—C6—C5	3.4 (11)	C18—C19—C20—O3	179.0 (8)
N3—C1—C6—C5	−176.9 (7)	C18—C19—C20—C15	−0.9 (12)
C7—O2—C6—C1	−0.2 (7)	C21—O3—C20—C19	−179.6 (7)
C7—O2—C6—C5	176.3 (7)	C21—O3—C20—C15	0.3 (7)
C4—C5—C6—C1	−1.1 (11)	C16—C15—C20—C19	2.3 (12)
C4—C5—C6—O2	−177.1 (7)	N6—C15—C20—C19	179.6 (7)
C1—N3—C7—N4	−178.6 (8)	C16—C15—C20—O3	−177.6 (7)
Ag1—N3—C7—N4	5.9 (11)	N6—C15—C20—O3	−0.3 (8)
C1—N3—C7—O2	−0.7 (8)	C15—N6—C21—N7	175.1 (7)
Ag1—N3—C7—O2	−176.2 (4)	C15—N6—C21—O3	0.1 (8)
C6—O2—C7—N3	0.6 (8)	C20—O3—C21—N6	−0.2 (8)
C6—O2—C7—N4	178.7 (7)	C20—O3—C21—N7	−175.9 (6)
C14—N1—C8—C13	2.2 (7)	C28—N8—C22—C23	179.3 (7)
Ag1—N1—C8—C13	171.8 (4)	C28—N8—C22—C27	−0.7 (7)
C14—N1—C8—C9	179.7 (7)	C27—C22—C23—C24	1.8 (10)
Ag1—N1—C8—C9	−10.7 (10)	N8—C22—C23—C24	−178.2 (7)
C13—C8—C9—C10	−0.9 (10)	C22—C23—C24—C25	−1.2 (11)
N1—C8—C9—C10	−178.1 (7)	C23—C24—C25—C26	−0.2 (12)
C8—C9—C10—C11	0.9 (12)	C24—C25—C26—C27	0.8 (11)
C9—C10—C11—C12	−0.3 (14)	C28—O4—C27—C22	−0.5 (7)
C10—C11—C12—C13	−0.3 (12)	C28—O4—C27—C26	−178.4 (7)
N1—C8—C13—C12	178.2 (7)	C23—C22—C27—O4	−179.3 (6)
C9—C8—C13—C12	0.3 (11)	N8—C22—C27—O4	0.8 (7)
N1—C8—C13—O1	−1.7 (7)	C23—C22—C27—C26	−1.3 (10)
C9—C8—C13—O1	−179.5 (6)	N8—C22—C27—C26	178.8 (6)
C11—C12—C13—C8	0.3 (11)	C25—C26—C27—O4	177.5 (7)
C11—C12—C13—O1	−179.9 (7)	C25—C26—C27—C22	−0.1 (11)
C14—O1—C13—C8	0.4 (7)	C22—N8—C28—N9	−180.0 (8)
C14—O1—C13—C12	−179.4 (7)	C22—N8—C28—O4	0.5 (8)
C8—N1—C14—N2	179.3 (8)	C27—O4—C28—N8	0.0 (8)
Ag1—N1—C14—N2	10.4 (11)	C27—O4—C28—N9	−179.6 (7)

### Bis(2-aminobenzoxazole-κN)silver(I) nitrate–bis(2-aminobenzoxazole (1/2) Hydrogen-bond geometry (Å, º)

*Cg*11 is the centroid of the C22–C27 ring.

**Table:**

*D*—H···*A*	*D*—H	H···*A*	*D*···*A*	*D*—H···*A*
N2—H2*A*···N6^i^	0.84 (2)	2.15 (5)	2.915 (8)	151 (7)
N2—H2*B*···O5	0.83 (2)	2.15 (3)	2.937 (10)	158 (8)
N2—H2*B*···O7	0.83 (2)	2.65 (7)	3.271 (10)	133 (8)
N4—H4*A*···O5	0.85 (2)	2.14 (4)	2.937 (9)	157 (9)
N4—H4*A*···O6	0.85 (2)	2.46 (6)	3.084 (8)	131 (6)
N4—H4*A*···N5	0.85 (2)	2.64 (4)	3.434 (8)	156 (7)
N7—H7*A*···O7^i^	0.84 (2)	2.17 (2)	3.012 (9)	175 (7)
N7—H7*B*···O6	0.85 (2)	2.24 (3)	3.069 (8)	165 (8)
N9—H9*A*···N8^ii^	0.85 (2)	2.18 (3)	2.997 (9)	161 (9)
N9—H9*B*···O6	0.85 (2)	2.28 (5)	3.041 (10)	149 (9)
N4—H4*B*···*Cg*11^iii^	0.85 (4)	2.58 (5)	3.403 (7)	162 (7)

Symmetry codes: (i) −*x*, −*y*+1, −*z*; (ii) −*x*, −*y*+1, −*z*+1; (iii) −*x*+1, −*y*+1, −*z*+1.

### pi-pi interactions

**Table:**

Cg(i)	Cg(j)	distance (Å)	dihedra anglel (°)
1	7^iv^	3.584 (4)	6.7 (4)
2	4^v^	3.609 (4)	2.5 (4)
4	7^iv^	3.953 (4)	6.4 (4)

Cg1 = O1-C13-C8-N1-C14; Cg2 = O2-C6-C1-N3-C7; Cg3 = C1-C6; Cg4 = C8-13; Cg7 = O3-C20-C15-N6-C21; Cg8 = C15-C20; Cg10 = O4-C27-C22-N8-C28; Cg11 = C22 -C27;Symmetry codes: (iv) x, y-1, z; (v) 1-x, -y, -z.
